# GOS Ameliorates Nonalcoholic Fatty Liver Disease Induced by High Fat and High Sugar Diet through Lipid Metabolism and Intestinal Microbes

**DOI:** 10.3390/nu14132749

**Published:** 2022-07-01

**Authors:** Shuting Qiu, Jiajia Chen, Yan Bai, Jincan He, Hua Cao, Qishi Che, Jiao Guo, Zhengquan Su

**Affiliations:** 1 Guangdong Engineering Research Center of Natural Products and New Drugs, Guangdong Provincial University Engineering Technology Research Center of Natural Products and Drugs, Guangdong Pharmaceutical University, Guangzhou 510006, China; q743160056@163.com (S.Q.); 15768996925@163.com (J.C.); 2 Guangdong Metabolic Disease Research Center of Integrated Chinese and Western Medicine, Key Laboratory of Glucolipid Metabolic Disorder, Ministry of Education of China, Guangdong TCM Key Laboratory for Metabolic Diseases, Guangdong Pharmaceutical University, Guangzhou 510006, China; 3 School of Public Health, Guangdong Pharmaceutical University, Guangzhou 510310, China; angell_bai@163.com (Y.B.); hejincan300@163.com (J.H.); 4 School of Chemistry and Chemical Engineering, Guangdong Pharmaceutical University, Zhongshan 528458, China; caohua@gdpu.edu.cn; 5 Guangzhou Rainhome Pharm & Tech Co., Ltd., Science City, Guangzhou 510663, China; cheqishi@rhkj.com.cn

**Keywords:** galactose oligosaccharide, nonalcoholic fatty liver disease, lipid metabolism, inflammation, intestinal microbes, prebiotics

## Abstract

The treatment of nonalcoholic fatty liver disease (NAFLD) remains very challenging. This study investigated the therapeutic effect of galactose oligosaccharide (GOS), an important prebiotic, on NAFLD through in vivo and in vitro experiments and preliminarily explored the mechanism by which GOS improves liver lipid metabolism and inflammation through liver and intestinal microbiological analysis. The results of mouse liver lipidomics showed that GOS could promote body thermogenesis in mice with high-fat and high-sugar diet (HFHSD)-induced NAFLD, regulate lipolysis in liver fat cells, and accelerate glycine and cholesterol metabolism. GOS dose-dependently reduced the contents of total cholesterol (TC) and triglyceride (TG) in cells and reduced the accumulation of lipid droplets in cells. GOS also reduced the Firmicutes/Bacteroidetes ratio and altered the composition of the intestinal microbiota in mice fed a HFHSD. GOS can improve liver lipid metabolism and intestinal structure of NAFLD. These results provide a theoretical and experimental basis supporting the use of GOS as a health food with anti-NAFLD functions.

## 1. Introduction

Nonalcoholic fatty liver disease (NAFLD) has become the most common chronic liver disease in most regions of the world. It is a cause of the high incidence of end-stage liver disease worldwide and an inducer of hepatocellular carcinoma (HCC) [1,2]. A study showed that the total prevalence of NAFLD in China from 1999 to 2018 was 29.6% (95% CI: 28.2–31.0%), and the total number of patients with NAFLD in China is expected to increase to 314.58 million by 2030 [3].

Hepatocyte death is one of the key trigger factors for liver inflammation in the progression of NAFLD and NASH. In addition, the intestine and liver are closely related. Recent studies have shown that the destruction of intestinal vascular barrier by microbial communities leads to the transfer of bacteria or bacterial products to the blood circulation, which is the premise of liver inflammation and NASH [4].

NAFLD is a major health problem affecting hundreds of millions of people around the world. To date, the Food and Drug Administration (FDA) has not approved a specific drug for NAFLD. Lifestyle interventions, including dietary habits and physical exercise, have become the first line of treatment for NAFLD and NASH [5].

Galactose oligosaccharide (GOS) is a functional food additive with the highest safety and natural properties among oligosaccharides. GOS is a well-known important prebiotic with optimal therapeutic effects and has been widely used in infant milk powder, fermented milk, bread, and other foods [6]. In recent years, due to the physicochemical properties and numerous physiological functions of GOS, more and more studies have focused on GOS. For example, GOS can regulate the balance of intestinal flora and improve lipid metabolism and mineral absorption [7,8]. Clinical studies [9] have shown that GOS (at least 12 g/d) can reduce appetite and food intake, and can decrease serum lipopolysaccharide (LPS) in a dose-dependent manner. GOS is a group of oligosaccharides with probiotic activity, although the exact health benefits of GOS remain to be studied.

This study investigated the therapeutic effect of GOS on NAFLD through in vivo and in vitro experiments, and the mechanism by which GOS improves liver lipid metabolism and inflammation from the perspective of liver and intestinal flora was preliminarily explored to provide a theoretical and experimental basis supporting the use of GOS as a health food with anti-NAFLD function.

## 2. Materials and Methods

### 2.1. Animal Experiments

#### 2.1.1. Animals

C57BL/6 male mice, aged 7 weeks, were purchased from Hunan SJA Laboratory Animal Co., Ltd. and raised in the Animal Experiment Center at Guangdong Pharmaceutical University. They were housed under specific pathogen-free (SPF) conditions, the ambient temperature of the experimental animal chamber was 24.0 ± 2.0 °C, the relative humidity was 54–65%, the number of air changes was >15 times/h, and the light/dark cycle was 12/12 h/day. All procedures were performed in accordance with the welfare standards of laboratory animals and were approved by ethical review (FAFU-2018-025).

#### 2.1.2. Establishment and Treatment of the NAFLD Mouse Model

Sixty healthy C57BL/6 male mice (7 weeks old) were fed an ordinary diet (provided by the Animal Experimental Center of Guangdong Pharmaceutical University). After 1 week of adaptive feeding, the mice were randomly divided into 2 groups. The blank control group (*n* = 10) was given ordinary feed. The second group was given a HFHSD (D12327) for 8 weeks and then randomly divided into 5 groups (10 in each group). At present, the maximum tolerance of dietary fiber is not clear. The maximum daily dose recommended by GOS at 45% concentration as a food supplement is 16.2 g, but the intake can reach 58 g after adding to the authorized food category [10]. This is according to the previous experiments of our research group and those previously published [11], such as the NAFLD model group (the model group), positive control group (metformin group; 50 mg/kg metformin), high-dose galactose oligosaccharide group (GOS-H; 2730 mg/kg GOS), medium-dose galactose oligosaccharide group (GOS-M; 1365 mg/kg GOS), and low-dose galactose oligosaccharide group (GOS-L; 681.5 mg/kg GOS).

**Figure 1:**
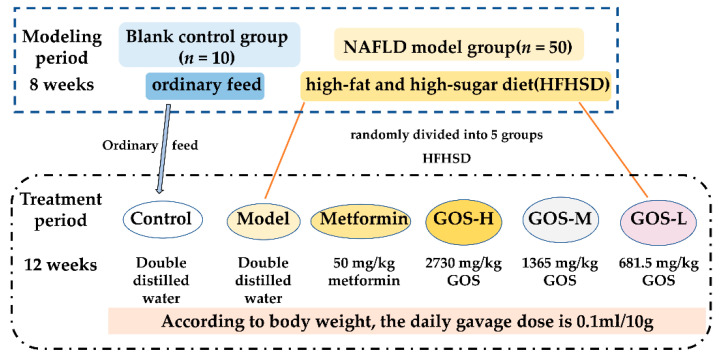
Experimental Design of NAFLD Mice.

The mice in metformin group and GOS group were given corresponding medicinal solutions solved in double distilled water by gavage daily according to body weight, and the mice in the control group and the model group were given double distilled water according to body weight (Figure 1). After 12 weeks of intervention, blood was collected by eyeball blood sampling, and the liver, cecal contents, feces, and other samples were collected and stored at −80 °C for subsequent use.

The normal feed was provided by Guangdong Provincial Medical Experimental Animal Center, and a HFHSD was purchased from Research Diets Company, D12327 (New Brunswick, NJ, USA. Metformin was provided by Sino-American Shanghai Squibb Pharmaceutical Co., Ltd. (Shanghai, China), and GOS was provided by New Froncisco (Yunfu, China). Biotechnology Co., Ltd (Yunfu, China). The specification of GOS is King-Prebiotics ^®^ 100% purity GOS. The content of galactooligosaccharides disaccharide is 0.8%, trisaccharide is 48%, tetrasaccharide is 30%, pentasaccharide is 20%, and hexasaccharide is 1.0%. The energy provided by normal feed was 3.4 Kcal/g, containing 23.07% protein, 65.08% carbohydrate, and 11.85% fat. The energy provided by HFHSD was 4.75 Kcal/g, containing 23% protein, 46.1% carbohydrate, and 20.4% fat.

#### 2.1.3. Biochemical Analysis

Analysis of serum indicators: The serum levels of TC, TG, HDL-C, LDL-C, ALT, AST, GLU, and FFAs were measured by ordinary kits (Jiancheng, Nanjing, China) (Table S1). The serum levels of insulin, TNF-α, IL-6, and IL-10 were measured by ELISA kits (Mei mian, Yancheng, China).

Analysis of liver indicators: The contents of TC, TG, HDL-C, and LDL-C in the livers of mice were measured with kits (Jiancheng, Nanjing, China). The contents of MDA, CAT, SOD, and GSH-Px were determined by ELISA kits (Mei mian, Yancheng, China).

#### 2.1.4. Glucose Tolerance Test

Six mice were randomly selected from each group (control, model, metformin, GOS-H, GOS-M, and GOS-L) for testing. The mice were fasted 12 h in advance but were provided water. The blood glucose of each mouse was measured at 0 min blood glucose level. According to the body weight of the mice, a glucose solution (2.0 g/kg) was intraperitoneally injected, and the blood glucose of each mouse at 15 min, 30 min, 60 min, 90 min, and 120 min was measured by Roche blood glucose meter.

#### 2.1.5. Histopathological Examination of Liver

The mice were dissected, their livers were weighed, and the liver index was calculated. Liver index = liver weight (g)/body weight (g). Livers were embedded and fixed in 4% PFA for 24 h. Hematoxylin–eosin (Leagene, Beijing, China) staining was performed. Finally, an electron microscope was used. The embedded frozen liver tissue was cut into 6 μm thick liver tissue sections with a frozen slicer. The liver tissue sections were stained with oil red O (Sigma-Aldrich, Shanghai, China) and observed with an optical microscope.

#### 2.1.6. Liver Metabolomics

The experimental samples were from the control group, model group, metformin group, and GOS-H group in animal experiments. After thawing, 50 mg of the samples was weighed and added to the corresponding 2 mL centrifuge tube to extract the liver tissue samples. Waters ACQUITY Ultra-performance liquid chromatography (UPLC) was used. UPLC and tandem mass spectrometry (MS/MS) were used to analyze the samples (UPLC, Shim-pack UFLC SHIMADZU CBM A system, https://www.shimadzu.com/, accessed on 21 September 2020; MS, QTRAP^®^ System, https://sciex.com/, accessed on 21 September 2020).

#### 2.1.7. Fecal Flora 16S rRNA Sequencing

Approximately 0.4 g (approximately 4 pieces) of fresh feces was taken from the GOS-H group, placed in a sterile centrifuge tube, rapidly frozen with liquid nitrogen, and stored at −80 °C for future use. Genomic DNA was extracted and amplified by PCR using a DNA extraction kit. The universal primers 515F and 806R were used to amplify the V3-V4 region of the bacterial 16S rRNA gene (Table S2). The 16S rRNA gene sequencing library of bacteria was prepared by using Thermo Fisher’s Ion Plus Fragment Library Kit 48 RXNS Library construction kit. After the constructed library was qualified by with a Qubit and library detection was performed, an Ion S5TMXL Thermo Fisher instrument was used for on-machine sequencing.

Using UPARSE software, clean reads were clustered into operational taxonomic units (OTUs) according to the consistency of paired nucleotide sequences with a threshold of 97%, and the sequences with the highest frequency in OTUs were considered representative OUT sequences. Based on the above analysis, alpha diversity analysis and beta diversity analysis were performed. In addition, *t* tests and linear discriminant analysis of effect size (LefSe) were used to identify significant differences in species among the groups to better understand the differences in bacterial communities between the groups.

#### 2.1.8. Analysis of Short-Chain Fatty Acids (SCFAs) in Feces

Fecal SCFAs were quantitatively analyzed by gas chromatography–mass spectrometry using an Agilent DB-FFAP capillary column (30 m × 25 mm × 0.25 μm) (Agilent Technologies, Santa Clara, CA, USA). The analyte was quantified using the target ion in the selected ion monitoring mode and confirmed by the confirmation ion.

#### 2.1.9. Fluorescence Quantitative PCR

Total RNA was extracted from liver tissue using RNAiso Plus reagent (Takara, Dalian, China), and the total RNA of each sample was reverse transcribed into cDNA using a reverse transcription kit with gDNA Eraser (Takara, Dalian, China). A reaction procedure was performed in a Light Cycler 480 fluorescence quantitative PCR instrument to generate DNA. After the reaction, the relative quantitative expression method 2^−^^ΔΔCt^ was used to compare the gene expression between groups, and the relative expression levels were calculated. The primers used in this study are listed in Table S3.

### 2.2. Cell Experiment

The hepatocellular carcinoma cell line HepG2 was purchased from the cell bank of the Shanghai Institute of Cell Biology, Chinese Academy of Sciences, and was preserved and passaged by our laboratory for future use. Oleic acid (OA) and sodium palmitate (PA) were purchased from Sigma-Aldrich.

GOS was fully dissolved in minimal medium DMEM (Thermo Fisher, Waltham, MA, USA), and the liquid was prepared by filtration and sterilization with a 0.22 μm microporous filter membrane before use; the mold liquid was made of 1mM FFA according to the ratio of OA:PA = 2:1.

#### 2.2.1. Model of Hepatocyte Steatosis

HepG2 cells in logarithmic growth phase were used for the experiment. The normal group cells were cultured with serum-free medium; the NAFLD group was cultured with 1 mM FFAs for 24 h. To determine the concentration of GOS to administer, cells were treated with different concentrations of GOS for 24 h. The CCK-8 method was used to determine the cell viability and calculate the cell survival rate (Meilun, Dalian, China).

#### 2.2.2. Cell Oil Red O Staining

HepG2 cells in the logarithmic growth phase were seeded on a 6-well culture plate at 1.2 × 10^6^ cells/well, and the cells were grouped according to the experiment, oil red O (Sigma-Aldrich, Shanghai, China) working solution was added for staining, and the cells were observed under a microscope.

#### 2.2.3. Media and Biochemical Analysis of Hepatocytes 

HepG2 cells in the logarithmic growth phase were seeded on a 6-well culture plate at 1.2 × 10^6^ cells/well, and the cells were processed in groups according to the experiment. The medium and hepatocytes were collected, and ALT and AST indexes were determined according to the instructions of the kit (Jiancheng, Nanjing, China).

#### 2.2.4. Fluorescence Quantitative PCR

As described in 1.2.6, after the reaction was completed, the relative quantitative expression method 2^−ΔΔCt^ was used to compare the gene expression between groups, and the relative expression levels were calculated. The primers used in this study are listed in Table S4 (Sangon, Shanghai, China).

### 2.3. Statistics and Analysis

The experimental data of this study were analyzed using GraphPad Prism 8.0 statistical software, and the experimental results are expressed as the mean ± SEM. A single factor was used to compare the averages of multiple groups of samples. A *p* value less than 0.05 indicates statistical significance. *T*-test was used for comparison between the two groups of data, and one-way ANOVA analysis was used for comparison of more than two groups of data. Image Pro Plus 6.0 software was used to calculate the ratio of oil red O staining lipid droplet area to total area. Uparse algorithm (Uparse v7.0.1001, http://www.drive5.com/uparse/, accessed on 17 October 2020) was used to cluster the sequences into OTUs. Qiime software (Version 1.9.1) was used to calculate Alpha Diversity. R software (Version 2.15.3) was used to draw PCA, PCoA, NMDS, and correlation clustering heat maps. LEfSe analysis uses LEfSe software, and the default LDA Score filter value is 4. We used KEGG database to annotate and display differential metabolites.

## 3. Results

### 3.1. The Effect of GOS on NAFLD Mice

#### 3.1.1. The Effect of GOS on the Body Weight and Food Intake of Mice

**Figure 2:**
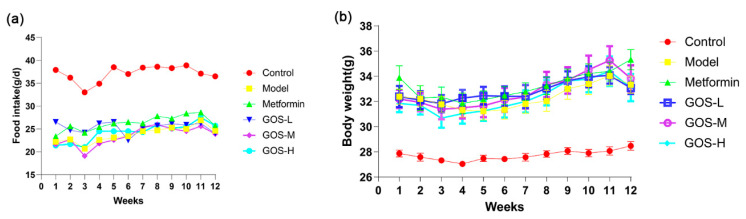
Average food intake of mice in each group during 12 weeks of administration (**a**) and weekly food intake of mice during the administration period (**b**), weekly body weight of mice changed after administration (*n* = 10, mean ± SEM).

The changes in food intake of mice in each group during the administration period were recorded. The results can be seen in Figure 2. There was no significant difference in the average weekly food intake and body weight of mice in the HFHSD-treated model group, metformin group, and GOS groups, indicating that GOS had no adverse effect on appetite inhibition. Weight gain is closely related to dietary structure. Studies have shown that the only reason for obesity in mice is that they consume more fat in their diet [12]. The HFHSD contains high fat, which is more conducive to the increase of body weight in mice.

#### 3.1.2. The Effect of GOS on Mouse Serum

**Figure 3:**
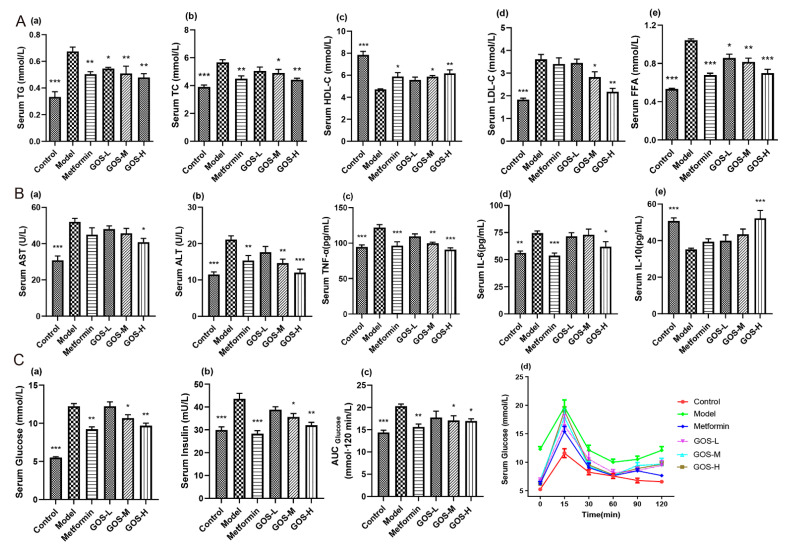
(**A**) Changes of TC (**a**), TG (**b**), HDL-C (**c**), LDL-C (**d**), and FFA (**e**) in serum of mice after administration (*n* = 10, mean ± SEM). (**B**) Changes in serum AST (**a**), ALT, (**b**) TNF-α (**c**), IL-6 (**d**), and IL-10 (**e**) of mice after administration (*n* = 10, mean ± SEM). (**C**) Changes of fasting blood glucose (**a**) and insulin in mice after administration (**b**) (*n* = 10, mean ± SEM). Changes in glucose tolerance of mice after administration (*n* = 6, mean ± SEM): (**c**) Blood glucose changes over time; (**d**) Area under GLU. Note: * *p* < 0.05, ** *p* < 0.01, *** *p* < 0.001 vs. the model group.

The effect of GOS on blood lipid metabolism are shown in Figure 3(Aa–e). After GOS administration in mice, compared with the model group, GOS could improve the dyslipidemia induced by HFHSD in mice, especially in the GOS-H group and GOS-M group. As shown in Figure 3(Ba,b), after GOS administration, the ALT and AST levels were notably decreased, and some doses had significant effects, indicating that GOS has a protective effect on the liver. As shown in Figure 3(Bc–e), compared with the model group, the GOS-H group significantly decreased the contents of TNF-α and IL-6 in the serum of mice and significantly increased the IL-10 level. GOS may protect the liver from secondary damage by reducing inflammatory infiltration in the late stage of HFHSD-induced liver injury in mice.

Glycemic control can predict the severity of balloon hepatocytes and liver fibrosis in NAFLD/NASH, so optimizing glycemic control may be a means to alter the risk of NASH-associated fibrosis progression [13]. As shown in Figure 3(Cc,d), each dose of GOS effectively alleviated the glucose tolerance in NAFLD model mice. As shown in Figure 3(Ca), the glucose concentrations in the GOS groups were lower than that in the model group, indicating that GOS has a certain hypoglycemic effect. Our animal experiments show that the long-term administration of a HFHSD in the model group resulted in a HOMA-IR index approximately three times that of the control group. As shown in Figure 3(Cb), the HFHSD induced insulin resistance in mice. The insulin content in the GOS treatment groups was lower than that in the model group. GOS can improve IR in mice. As shown in Figure 3(Cc,d), each dose of GOS effectively alleviated glucose tolerance in NAFLD model mice.

#### 3.1.3. The Effect of GOS on the Mouse Liver

**Figure 4:**
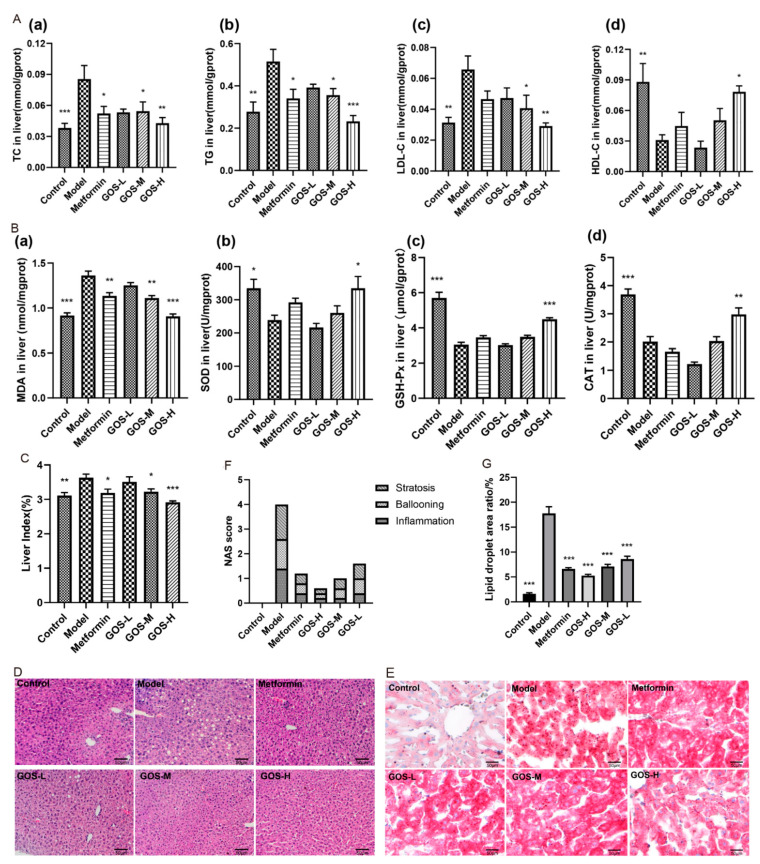
(**A**) Changes in the levels of TC (**a**), TG (**b**), LDL-C (**c**), and HDL-C (**d**) in the liver of rats after administration. (**B**) Changes in the levels of MDA (**a**), SOD (**b**), GSH-Px (**c**), and CAT (**d**) in the liver of mice after administration. (**C**) Mice liver index after administration. (**D**,**E**) H&E staining and oil red O staining (200×, scale bar 50 μm) (*n* = 10, mean ± SEM). (**F**) Mice NAS scoring (*n* = 5). (**G**) The ratio of lipid accumulation area to total liver area in the liver (*n* = 5, mean ± SEM). Note: * *p* < 0.05, ** *p* < 0.01, *** *p* < 0.001 vs. the model group.

NAFLD is a lipotoxic disease in which liver steatosis and oxidative stress are key pathogenic features. The liver lipid content of mice in each group is shown in Figure 4(Aa–d). After GOS administration, the GOS-H group and GOS-M group showed significantly reduced liver lipid levels in HFHSD-fed mice and improved liver steatosis. 

Figure 4(Ba–d) shows that after GOS administration, the oxidative damage index of the mouse liver was improved, thereby reducing the degree of liver damage. Liver index can represent the degree of liver injury. The larger the index, the larger the liver lipid accumulation. The liver index in the model group increased significantly, suggesting that liver lipid accumulation was serious. As shown in Figure 4C, the liver weight and liver index in the control, GOS-H, and GOS-M groups were significantly lower than those in the model group, indicating that GOS can improve HFHSD-induced liver hypertrophy in mice. The overall morphology of the liver of mice was observed. The results of H&E staining and oil red O staining of mouse liver also confirmed this point, as shown in Figure 4D,E. In Figure 4D there was no obvious abnormality in liver cells of mice in the control group, and there were a large number of lipid droplets in mice in the model group, and the lipid droplets in the GOS administration group were significantly reduced. Five high-power microscopic fields were randomly observed under the microscope, and liver lesions were scored according to NAS scoring criteria (Figure 4F). The scoring criteria are shown in Table S5. In Figure. 3E, the lipid droplets in the model group were stained red, and the lipid content in the liver cells of the GOS administration group decreased. Image Pro Plus 6.0 software was used to calculate the ratio of lipid accumulation area to total liver area. Compared with the model group, GOS group reduced the ratio of lipid droplet area in mouse liver (Figure 4G). This result indicated that GOS reduced HFHSD-induced lipid infiltration and improved hepatic steatosis in mice.

#### 3.1.4. Mouse Liver Lipidomics

**Figure 5:**
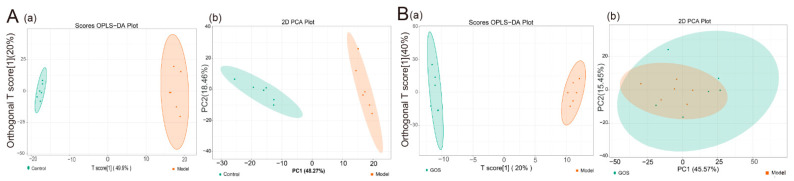
(**A**) OPLS-DA score plot (**a**) and PCA plot (**b**) of liver tissue samples from the Control group and the Model group; (**B**) OPLS-DA score plot (**a**) and PCA plot (**b**) of liver tissue samples from the GOS group and the model group.

Combined with the beneficial effects of GOS on the serum and liver of mice, the liver samples of the control group, model group, metformin group, and GOS-H group mice in animal experiments were selected for analysis. Figure 5 shows significant changes in the abundance of metabolites in the liver of HFHSD-fed mice compared with normal diet mice. Multivariate statistical analysis showed that GOS could improve the liver sample composition of HFHSD-induced mice. Figure 5 shows that GOS improves HFHSD-induced liver metabolite abundance in mice.

**Figure 6:**
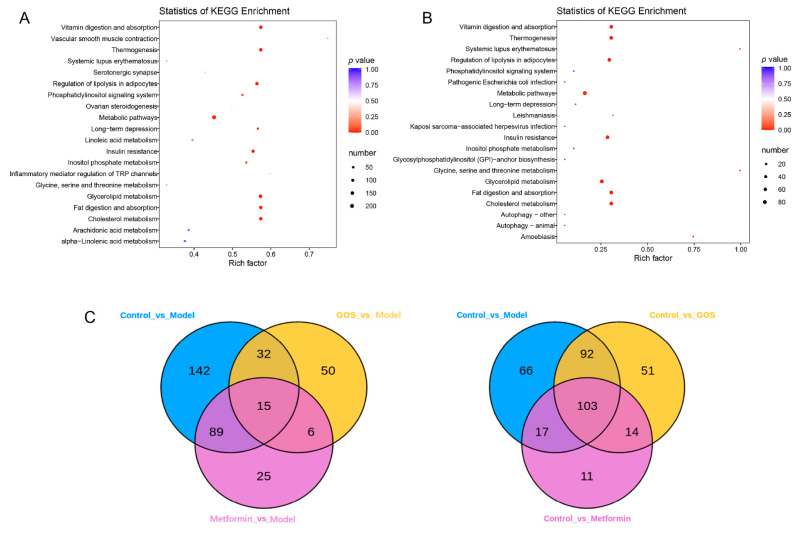
(**A**) Metabolite KEGG enrichment diagram of liver tissue samples in control and model. (**B**) Metabolite KEGG enrichment diagram of liver tissue samples in GOS and model. Venn diagram of metabolites in different groups where the Rich Factor was the ratio of the number of metabolites in the corresponding pathway to the total number of metabolites annotated by the detection of that pathway. The larger the value was, the greater the enrichment degree was. (**C**) Venn diagram of metabolites in different groups.

The differential metabolites of control group and model group in mice were analyzed. The comparison of ion fragment levels in the control and model groups was based on OPLS-DA analysis and filtering, combined with *t* test (*p* < 0.05) and fold change (FC) ≥ 2 conditions, and accurate fragments through high-resolution mass spectrometry retention time (Rt) and mass-to-charge ratio (*m*/*z*) of precursor ions and product ions were imported into a local database to search and determine the specific structural properties of potential biomarkers. After the above screening, a total of 278 different metabolites were found, of which 163 were downregulated and 114 metabolites were upregulated in the model group compared with the control group (see Table S6, Figure 6, and Supplementary File S1). The above data suggest that the main HFHSD-induced molecular perturbations of the metabolic phenotype of mice are triglycerides, phosphatidylcholine, diglycerides, and eicosanoids. KEGG pathway enrichment analysis of the differential metabolites was carried out. As shown in Figure 6A, it is clear that the HFHSD-induced differential metabolites in mice are mainly enriched in the vitamin digestion and absorption, thermogenesis, lipid decomposition regulation in fat cells, insulin resistance, and glycine and cholesterol metabolism.

The differential metabolites between the GOS group and model group were analyzed. After the above qualitative screen, a total of 103 different metabolites were identified in the GOS group and model group, of which 21 were downregulated and 82 were upregulated in the GOS group compared with the model group (see Supplementary File S2). The above data suggest that GOS can improve lipid metabolism in mice with HFHSD-induced NAFLD, mainly by changing the abundance of triglycerides, phosphatidylserine, diglycerol, lysophosphatidylcholine, and phosphatidylethanolamine species. It can be seen intuitively from Figure 6B that GOS treatment corrected the dysregulation of cholesterol and triglyceride metabolism and improved the insulin resistance induced by the HFHSD in mice. As shown in Table S6 and Figure 6, these results indicated that different diets and treatments had different effects on mice, and the expression of metabolites in mouse livers was different.

#### 3.1.5. The Effect of GOS on the mRNA Expression Levels in Mouse Liver

**Figure 7:**
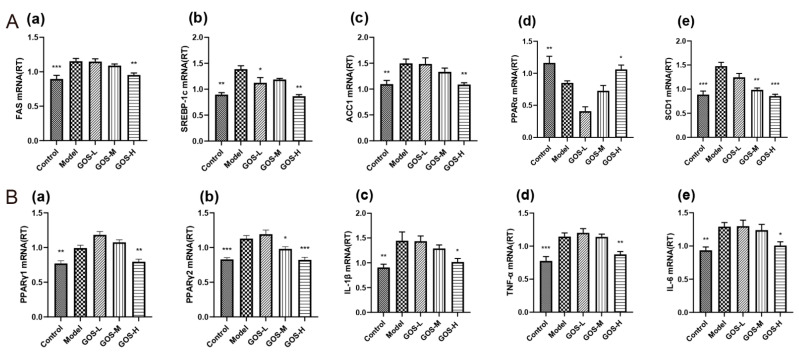
(**A**) Effects of GOS on the expression levels of FAS (**a**), SREBP-1C (**b**), ACC1 (**c**), PPARα (**d**), and SCD1 (**e**) genes in mice liver (*n* = 6, mean ± SEM). (**B**) Effects of GOS on the expression of PPARγ1 (**a**), PPARγ2 (**b**), IL-1β (**c**), TNF-α (**d**), and IL-6 (**e**) genes in mice liver (*n* = 6, mean ± SEM). Note: * *p* < 0.05, ** *p* < 0.01, *** *p* < 0.001 vs. the model group.

To clarify the potential mechanism of GOS, the liver mRNA expression levels of genes involved in lipid metabolism and inflammation were determined by PCR. The main source of liver lipid synthesis is the de novo synthesis of lipids. SREBP-1c is closely related to the regulation of fatty acid and cholesterol synthesis and binds to the endoplasmic reticulum. As shown in Figure 7(Aa–c), after GOS treatment, GOS reduced the high expression of SREBP-1c, FAS, and ACC1, and inhibited the de novo synthesis of lipids.

Fatty acid oxidation in the liver is an important pathway affecting the development of fatty liver. As shown in Figure 7(Ad), the expression of the PPARα gene in the liver in the model group compared with the control group suggested that the HFHSD blocked the lipid oxidation pathway in the NAFLD mice. Additionally, compared with the model group, the GOS-H group showed significantly increased PPARα expression. Another important fatty acid metabolic pathway is the formation of triglycerides through esterification. As shown in Figure 7(Ae), the content of SCD-1 in each group decreased after GOS administration. These results indicated that GOS could exert its lipid-lowering effect by regulating lipid oxidation.

The lipid export-related gene PPARγ was significantly increased in the model group fed the HFHSD compared with the control group, as shown in Figure 7(Ba,b). GOS administration reduced the expression of PPARγ in the livers of mice, indicating that GOS can promote liver lipid export.

IL-1β and IL-6 are important inflammatory mediators in many inflammatory diseases. Figure 7(Bc–e) shows that the long-term administration of the HFHSD led to a significant upregulation in inflammatory gene expression, and GOS had a significant inhibitory effect on inflammatory gene expression. It is suggested that GOS can inhibit the body’s inflammatory response, thereby protecting the liver.

#### 3.1.6. The Effect of GOS on the mRNA Expression Levels in Mouse Liver

##### 16S rRNA Sequencing Data Processing and Analysis

**Figure 8:**
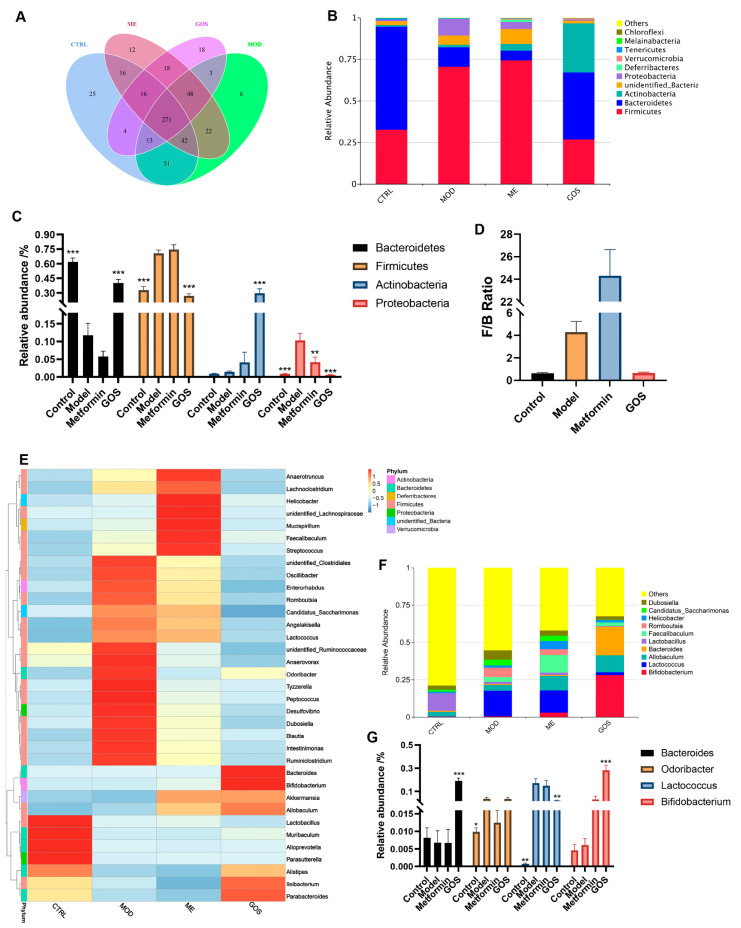
(**A**) Venn diagram of OTUs sequence of stool sample. (**B**) Relative abundance of species at the bacteria Phylum level. (**C**) The relative abundance statistics of the largest species in each group after administration at the bacteria Phylum level. (**D**) The effect of GOS on the relative abundance ratio of Firmicutes and Bacteroides. (**E**) Heat map of relative abundance of different metabolites in each group at the bacteria Phylum level. (**F**) Relative abundance of species at the bacteria genus level. (**G**) The relative abundance statistics of the largest species in each group after administration at the bacteria genus level. Note: * *p* < 0.05, ** *p* < 0.01, *** *p* < 0.001 vs. the model group (*n* = 4–5, mean ± SEM).

To explore the potential role of intestinal flora in NAFLD induced by a HFHSD and the effect of intestinal flora on NAFLD after GOS treatment, we performed 16S rRNA sequencing on the feces of mice treated with high-dose GOS in the experiment. DNA sequencing was performed on 20 fecal samples. The obtained valid sequences were clustered into OTUs according to 97% identity, and a total of 567 OTUs were obtained: 445 in the control group, 458 in the model group, 451 in the metformin group, and 387 in the GOS group, as shown in Figure 8A. The petal diagram shows the number of common and unique bacteria.

##### Analysis of GOS on the Relative Abundance of Intestinal Microbial Species in NAFLD Mice

According to the OTU annotations, the four groups of mouse fecal flora were analyzed for community composition at the phylum and genus levels. Generally, species with an abundance >1% can be defined as the dominant species. The results of the phylum-level analysis shown in Figure 8B,C show that the most abundant phyla in the mouse fecal flora of the four groups are *Firmicutes*, *Bacteroidetes*, and *Actinobacteria*, which are the dominant bacteria in the mouse intestinal flora. Compared with the control group, the model group showed significantly increased abundances of *Firmicutes* and *Proteobacteria* (*p* < 0.0001, *p* < 0.001) and significantly decreased abundance of *Bacteroidetes* (*p* < 0.0001). Compared with the model group, the GOS group had significantly upregulated abundances of *Bacteroidetes* and *Actinobacteria* (*p* < 0.0001, *p* < 0.0001) and downregulated abundance of *Firmicutes* and *Proteobacteria* (*p* < 0.0001, *p* < 0.001). After administration of GOS treatment, *Firmicutes* levels were reduced to normal, close to the level of healthy mice. As shown in Figure 8D, compared with the model condition, GOS intervention significantly reduced the F/B ratio (*p* < 0.05), indicating that GOS can reverse the changes in the structure of the intestinal flora induced by the HFHSD.

For the genus level analysis, Figure 8E shows many pathogenic bacteria (such as inflammation-related *Desulfovibrio* and *Tyzzerella*) that were significantly upregulated in the model group, and *Bifidobacterium* was also significantly upregulated in the GOS group. The gut microbes of the four groups were analyzed. The top ten most abundant genera were *Odoribacter*, *Lactobacillus*, *Bacteroides*, *Lactococcus*, *Dubosiella*, *Bifidobacterium*, *Erysipelatoclostridium*, *Allobaculum*, *Ileibacterium*, and *Feacalibaculum*. “Others” represents the sum of the abundance of bacteria of other genera outside the top 10 genera. Among them, *Bifidobacterium* and *Bacteroidetes* were significantly upregulated in the GOS group compared with the model group (*p* < 0.0001, *p* < 0.0001), and *Lactococcus* was significantly downregulated (*p* < 0.01). The specific statistics are shown in Figure 8F,G. Compared with the control condition, the GOS intervention reversed the influence of the HFHSD on the abundance of *Lactococcus*, *Bacteroides*, and *Bifidobacterium*.

##### Effect of GOS on the Diversity of Intestinal Microbial Communities in NAFLD Mice

**Figure 9:**
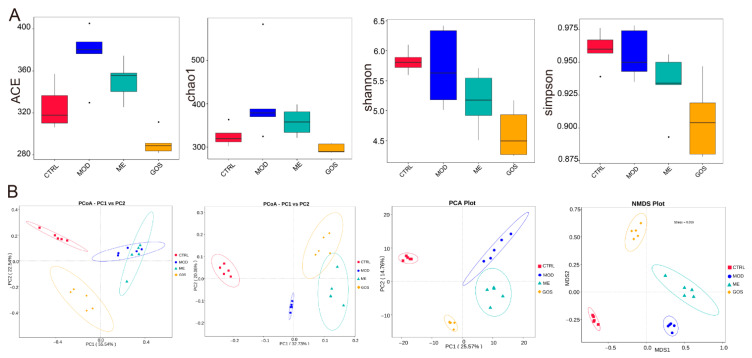
(**A**) Comparison of differences between groups of alpha diversity index. (**B**) Dimensionality reduction analysis results of beta diversity of mice in each group.

In this study, alpha diversity analysis was performed based on OTU clustering of the four groups of mouse fecal flora. The results are shown in Figure 9A. It can be seen that GOS intervention can reverse the increase in the abundance of intestinal microbial communities caused by the HFHSD to a certain extent, and at the same time reduce the diversity of the intestinal microbiota of mice, decreasing complexity of the sample community. After GOS intervention, each sample was also separated from the model group sample as a whole.

##### Analysis of the Effect of GOS on Different Species of Intestinal Microbial Colonies in NAFLD Mice

**Figure 10:**
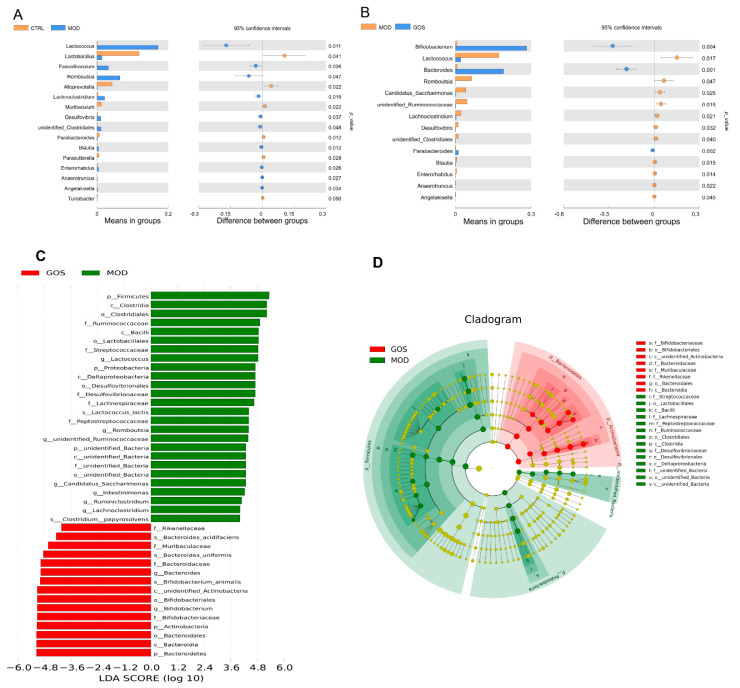
Species difference analysis diagram between *t*-test groups: (**A**) The control group vs the model group (*n* = 5), (**B**) the model group vs the GOS group (*n* = 5). (**C**) Histogram of LDA value distribution (*n* = 5), (**D**) LEfSe analysis evolutionary branch diagram (*n* = 5).

To study the different species between the groups at the genus level, a t test between groups was carried out. As shown by the results in Figure 10A,B compared with the model group, the GOS group showed significantly upregulated levels of *Bifidobacterium*, *Bacteroides*, and *Parabacteroides* in the fecal samples. Additionally, GOS treatment significantly reduced the levels of *Lactococcus*, *Romboutsia*, *Candidatus_Saccharimonas*, *unidentified_Ruminococcaceae*, *Lachnoclostridium*, *Desulfovibrio*, *unidentified_Clostridiales*, *Blautia*, *Enterorhabdus*, and other bacterial species. It can be seen that the GOS intervention can significantly reduce the abundance of a series of harmful bacteria, thereby improving the structure of the intestinal flora and treating NAFLD.

We further used the LEfSe algorithm and linear discriminant analysis (LDA) to analyze the species with differences in abundance between the model group (*n* = 5) and the GOS group (*n* = 5). The types of dominant flora are shown in Figure 10C. Among the marker flora with LDA values >4, 26 species were significantly enriched in the model group, and 15 species were significantly enriched in the GOS group. The p, c, o, f, g, and s in front of each group represent the phylum, class, order, family, genus, and species of the species, respectively. Figure 10D shows the evolutionary clade obtained from the LEfSe analysis. The results of this analysis showed that at the phylum level, GOS intervention significantly enriched *Bacteroidetes* and *Actinobacteria*, and *Firmicutes*, *Proteobacteria*, and *unidentified_Bacteria* were significantly enriched in the model group.

##### The Effect of GOS on Short-Chain Fatty Acids in the Cecal Contents of NAFLD Mice

**Figure 11:**
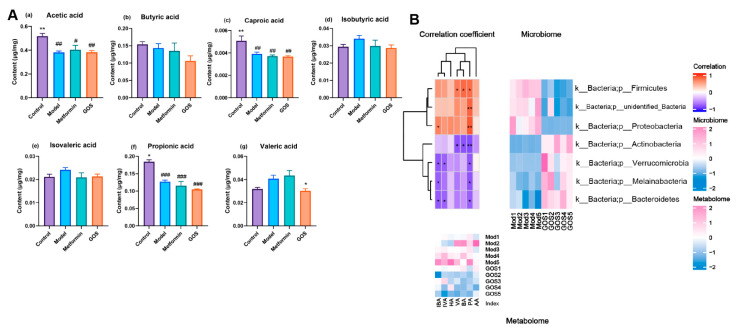
(**A**) Changes of SCFAs in the cecum content of mice in each group. Note: * *p* < 0.05, ** *p* < 0.01 vs. the model group; # *p* < 0.05, ## *p* < 0.01, ### *p* < 0.001 vs. the control group (*n* = 4–5, mean ± SEM). (**B**) Heat map of Spearman correlation at the phylum level. The strength of the color represents the degree of association. The heatmap shows the Spearman correlation between different microorganisms and different metabolites. The abscissa represents metabolites, the ordinate represents microorganisms, * represents a *p* value of < 0.05 in the correlation coefficient significance test, and ** represents a *p* value < 0.01. The abundance data of microorganisms and metabolites were standardized using Z scores.

In addition to intestinal microbial disruption, metabolites related to the intestinal flora, such as SCFAs, may have an impact on the pathogenesis of NAFLD and play an important role in regulating the balance of intestinal microbes and improving body metabolism [14]. Figure 11A shows that compared with the model group, the GOS group had significantly downregulated valeric acid (VA) contents (*p* < 0.05). Compared with the control group, GOS significantly decreased the concentrations of acetic acid (AA), caproic acid (HA), and propionic acid (PA). Long-term supplementation with high-dose GOS may have a negative impact on the glucose metabolism of the host, which suggests that we need to optimize the method of GOS administration. Additionally, GOS reversed the changes in isovaleric acid (IVA) and isobutyric acid (IBA) contents induced by the HFHSD, but there was no significant difference.

##### Correlation between Intestinal Flora and Metabolites

The intestinal flora is closely related to the body’s metabolism, and SCFAs, a byproduct of microbial metabolism, are an important source of energy for the body and have a regulatory effect on the body’s energy metabolism. Figure 11B shows the results of a correlation analysis between the differential microorganisms and differential metabolites detected in each group. A correlation heatmap obtained by calculating the Spearman correlation coefficients of microorganisms and metabolites shows the correlation between the different microorganisms and the different metabolites at the phylum and genus levels in each group.

Spearman correlation hierarchical cluster analysis was performed on the gut microbes and their metabolites in the model and GOS groups. Figure 11B shows that at the phylum level, IBA was significantly positively correlated with *Proteobacteria* and significantly negatively correlated with *Verrucomicrobia*, *Melainabacteria*, and *Bacteroidetes*. IVA was significantly negatively correlated with *Verrucomicrobia*, *Melainabacteria*, and *Bacteroidetes*. Propionic acid (PA) was significantly positively correlated with *Firmicutes*, *unidentified_Bacteria*, and *Proteobacteria*, and significantly negatively correlated with *Actinobacteria*, *Verrucomicrobia*, *Melainabacteria*, and *Bacteroidetes*. Both VA and BA were significantly positively correlated with *Firmicutes* and significantly negatively correlated with *Actinobacteria*. 

### 3.2. The Effect of GOS on Fatty Degeneration of HepG2 Cells

#### 3.2.1. GOS Reduces FFA-Induced HepG2 Hepatocyte Damage

**Figure 12:**
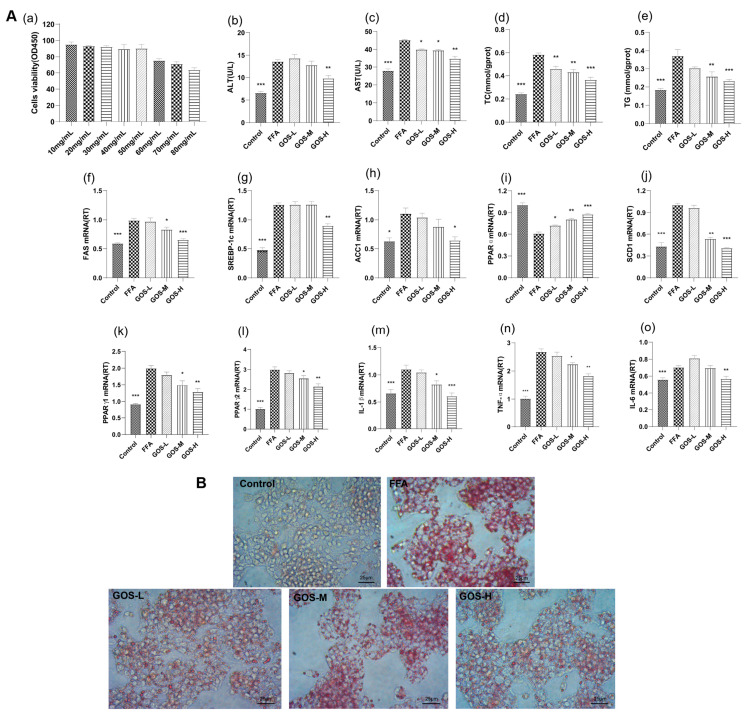
(**A**) (**a**) Cell survival rates under different GOS concentrations (*n* = 6, mean ± SEM). ALT (**b**) and AST (**c**) contents in cell culture medium, intracellular TC (**d**) and TG (**e**) contents in each group after administration (*n* = 6, mean ± SEM). Effects of GOS on the expression of FAS (**f**), SREBP-1c (**g**), ACC1 (**h**), PPARα (**i**), SCD1 (**j**) PPARγ1 (**k**), PPARγ2, (**l**) IL-1β (**m**), TNF-α (**n**), and IL-6 (**o**) genes in each group (*n* = 6, mean ± SEM). Note: * *p* < 0.05, ** *p* < 0.01, *** *p* < 0.001 vs. the FFA group. (**B**) Oil red O staining of cells in each group (400×).

GOS concentration was analyzed by CCK-8 assay. The results are shown in Figure 12(Aa). With a survival rate higher than 90%, the standard, high, medium, and low doses of GOS were selected for follow-up experiments. The low, medium, and high concentrations of GOS selected were 10 mg/mL, 20 mg/mL, and 30 mg/mL, respectively. The results are shown in Figure 12(Ab,c). Under the combined action of GOS, the GOS-H group showed significantly reduced ALT and AST contents in the culture medium. This shows that GOS can reduce the hepatocyte damage induced by FFAs to a certain extent.

#### 3.2.2. GOS Reduces FFA-Induced Degeneration of HepG2 Hepatocytes

As shown in Figure 12(Ad,e), compared with the FFA group, each GOS administration group had significantly reduced TG and TC contents. The optical microscope observation results of oil red O staining are shown in Figure 12B and Table S7. In the FFA group, after culturing with FFA for 24 h, liver cells with fatty degeneration were observed, accompanied by lipid droplet fusion. The administration group showed reduced lipid droplet accumulation in the cells, and as the concentration of GOS administration increased, the effect became more obvious.

#### 3.2.3. The Effect of GOS on the Lipid Metabolism Indexes of HepG2 Cells

Figure 12(Af–h) shows that after GOS intervention, GOS reduced the high expression of FAS, SREBP-1c, and ACC1 and inhibited the de novo synthesis of lipids. Figure 12(Ai,j) shows that, compared with the control condition, FFA stimulation reduced the expression of PPARα in the cells and increased the expression of SCD1. GOS intervention significantly increased the expression of PPARα and SCD1. Figure 12(Ak,l) shows that GOS intervention reduced the increased expression of PPARγ and accelerated lipid export. As shown in Figure 12(Am–o), after GOS intervention, the expression of inflammatory factors was reduced, protecting cells from damage.

Through cell experiments, it was found that GOS can reduce FFA-induced lipid deposition in HepG2 hepatocytes and reduce the expression of inflammatory factors, which was consistent with the results of the above animal experiments.

## 4. Discussions

This article proves that 8 weeks of HFHSD feeding can cause hyperglycemia, IR, dyslipidemia, liver damage, and liver steatosis, and successfully establish a mouse NAFLD model. GOS can protect liver cells and improve liver function in the early stages of liver cell damage caused by liver lipid deposition. GOS can improve IR, an important feature of metabolic syndrome in mice, and a safe and reliable hypoglycemic. GOS can also relieve oxidative stress in mice and reduce the expression of inflammatory factors.

Therefore, our research suggests that GOS can improve NAFLD in mice. Its therapeutic mechanism includes the abovementioned multiple signaling molecules and transduction mechanisms. These findings indicate that GOS is a promising drug for the treatment of NAFLD. Based on this, this experiment further studied the mechanism by which GOS improves NAFLD in mice.

Nontargeted lipidomics was used to analyze the changes in different lipid metabolites in the liver tissues of each group of mice and the related lipid metabolism pathways and to further explore the possible mechanism of GOS in the treatment of NAFLD caused by HSHFD from the perspective of lipid metabolism. The lipidomics analysis of mouse liver showed that, compared with the livers of mice fed a normal diet, the livers of mice fed the HFHSD showed significant changes in the abundance of metabolites. GOS promotes the thermogenesis of NAFLD mice, and the crosstalk mechanism between different cells changes the microenvironment of heat production, which has therapeutic potential for NAFLD [15]. Dysregulation of lipid metabolism will lead to excessive production of fatty acids (FA), thereby changing the metabolic pathways of adipose tissue and liver [16]. GOS regulates the lipid decomposition of adipocytes in the liver and promotes insulin secretion [17]. Supplementation of glycine reduces systemic glucose, lipid, and steatohepatitis [18]. The damage of cholesterol homeostasis in liver is closely related to NAFLD [19]. Dietary cholesterol can drive the formation of NAFLD-HCC by changing the intestinal microflora and its metabolites in mice after intestinal absorption [20]. GOS regulates the metabolism of glycine and cholesterol in NAFLD mice, which has a certain therapeutic potential.

GOS changed the abnormal lipid metabolism in NAFLD mice by regulating lipid synthesis and metabolism. When the synthesis, transport, and decomposition of fatty acids and TGs in the liver are hindered, lipids may be deposited in the liver and develop fatty liver through complex steps [21]. In our experiments, we detected changes in lipid synthesis and decomposition pathways in the livers of mice, as well as changes in inflammation pathways. In addition to in vivo experiments, normal human liver cancer cell line HepG2 was cultured in vitro, and the model was stimulated by FFA, which successfully damaged the HepG2 hepatocytes. PCR experiments explored the potential mechanism of GOS. It was found that it could inhibit the expression of SREBP-1c, FAS, and ACC1 involved in de novo lipid synthesis pathway, reduce the expression of PPARγ1 and PPARγ2 involved in liver lipid synthesis, and increase the expression of PPARα involved in liver lipid oxidation. It also inhibits the expression of TNF-α, IL-6, and IL-1β to exert an anti-inflammatory effect.

Long-term intake of foods rich in lipids and sugars can lead to increased intestinal nutrient uptake, resulting in abnormal lipid accumulation in the liver, adipose tissue, and other organs. For a long time, gut microbiota and host maintained a symbiotic relationship. In terms of intestinal microbes, after GOS intervention, the levels of *Bifidobacterium*, *Bacteroides*, and *Parabacteroides* were significantly increased. GOS decreased the levels of genera related to metabolism, such as *Lactococcus*, *Lactobacillus*, and *Desulfovibrio*. *Bacteroides* can inhibit the reproduction of harmful bacteria in the intestines, has an anti-inflammatory effect, can maintain the balance of intestinal flora while improving host metabolism, and has an important role in reducing fat and promoting weight loss [22,23,24]. *Parabacteroides* has an active role in regulating the body’s metabolism and can maintain the health of the intestinal barrier [25,26]. *Bifidobacterium* and *Lactococcus*, common probiotics, can enhance the intestinal epithelial barrier [27,28]. *Desulfovibrio* produces inflammatory factors and has a proinflammatory effect [29,30], and *unidentified_Ruminococcaceae* is related to metabolic disorders [31]. Therefore, GOS may use these beneficial bacteria to promote weight loss, maintain intestinal barrier health, improve insulin sensitivity, inhibit inflammation, and control blood sugar in mice. Moreover, GOS administration reduces the abundance and diversity of intestinal microbes. Although GOS increases beneficial bacteria and reduces harmful bacteria, it is important to pay attention to the possible negative effects of decreased abundance and diversity of intestinal microbes. At the phylum level, *Bacteroidetes* and *Firmicutes* have anti-inflammatory effects and participate in the regulation of host lipid metabolism. An increased *F/B* ratio may indicate a state of inflammation in the mouse intestine. The GOS intervention reversed the increase in the *F/B* ratio observed in the model group, suggesting that GOS may improve inflammation in the liver through changes in intestinal microbes.

SCFAs are the key bridge connecting intestinal microorganisms and the body. In the Spearman correlation hierarchical clustering analysis of intestinal flora and metabolites, the levels of *Firmicutes*, *unidentified_Bacteria*, and *Proteobacteria* in the model and GOS groups were positively correlated with the production of SCFAs. Additionally, the levels of *Actinobacteria*, *Verrucomicrobia*, *Melainabacteria*, and *Bacteroidetes* and the production of SCFAs were negatively correlated. Acetic acid, PA, and BA are the most abundant SCFAs in the intestinal tract and can lower the pH of the intestinal tract, enrich beneficial bacteria, and inhibit the growth of harmful bacteria [32]. Acetic acid has an anti-inflammatory effect, and PA can interact with intestinal fatty acid receptors to regulate the gluconeogenesis pathway [33]. BA can supply energy to intestinal epithelial cells, improve insulin resistance, reduce inflammation, and maintain intestinal homeostasis, but high concentrations of BA can aggravate damage to intestinal barrier function [34,35,36]. *Bacteroidetes* mainly produce acetic acid and PA, and *Firmicutes* mainly produces BA. IBA is produced by the metabolism of *Bifidobacterium*, which can improve insulin sensitivity by regulating fat cell lipid and glucose metabolism. However, the production of both IBA and IVA is accompanied by the production of harmful substances such as indole and amines [37]. Compared with the model group, GOS treatment reduced the contents of VA, IBA, and IVA to be similar to those in the control group. Additionally, GOS treatment reduced the contents of BA, PA, and HA, which may be the result of the synergistic effect of different bacterial genera. The decrease in SCFA content is consistent with the decrease in the abundance and diversity of intestinal flora. This suggests that GOS can be used to modulate intestinal microbes, which can bring certain beneficial effects on the host by adjusting the structure of intestinal microbes and their metabolites. Although the beneficial effects of GOS on the intestinal tract are consistent with the results of liver lipidomics studies, there were some unexpected results, such as the decrease in BA content in the intestinal tract of mice.

In recent years, a large number of studies have shown that intestinal flora disorders are closely related to NAFLD [38,39]. In addition, the incidence of NAFLD in obese patients is as high as 75% [40], so it is speculated that NAFLD may be closely related to obesity. Obesity-related genes (fat mass- and obesity-associated genes, FTOs) can increase body mass index and body fat [41]. In the future, we can further evaluate whether different doses of GOS can reduce the fat content in the body by regulating the intestinal flora and forming the intestine–liver–fat axis pathway, thus playing a role in the treatment of NAFLD.

## 5. Conclusions

**Figure 13:**
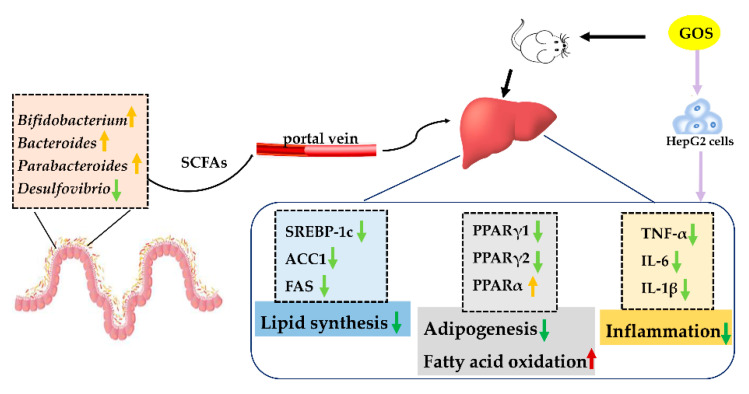
The mechanism of GOS improving liver lipid accumulation in mice. Light green arrows indicate downregulation, green arrows indicates decrease, yellow and red arrows indicate an increasing effect.

In this study, non-targeted metabolomics technology was used to preliminarily explore the mechanism of GOS improving liver lipid accumulation in mice, which may be related to inhibiting lipid synthesis, promoting oxidative decomposition, and reducing the expression of inflammatory factors (Figure 13). Additionally, GOS increases the levels of beneficial bacteria, reduces the reproduction of harmful bacteria, and has a beneficial effect on the liver by regulating the structure of the intestinal flora and its metabolites. The above results indicate that GOS can be developed as a health care product and medicine for the treatment of NAFLD.

## Data Availability

The data presented in this study are available on request from the corresponding author.

## Supplementary Materials

The following supporting information can be downloaded at: https://www.mdpi.com/article/10.3390/nu14132749/s1, Supplementary File 1, Differential metabolites in mice in the control group and model group; Supplementary File 2, Differential metabolites between the GOS group and model group mice; Table S1, Reagent and Company; Table S2, Primer information of the V3-V4 region of the bacterial 16S rRNA gene; Table S3, Primer sequences of animal experiment; Table S4, Primer sequences of cell experiment; Table S5 Non-alcoholic fatty liver disease activity score (NAS) scoring criteria; Table S6, Statistical table of the number of different metabolites in each group; Table S7, OD values of each group stained with oil red O (*n* = 6, mean±SEM).
