# Supplementary material for: GOS Ameliorates Nonalcoholic Fatty Liver Disease Induced by High Fat and High Sugar Diet through Lipid Metabolism and Intestinal Microbes
Source: Nutrients. 2022 Jul 1;14(13):2749. doi: 10.3390/nu14132749 (PMC9268751; doi:10.3390/nu14132749)
Supplement: Supplementary file 1 [file nutrients-14-02749-s001.zip › nutrients-1780037-supplementary.pdf]

Table S1 Reagent and Company

Reagent	Company
ALT kits、AST kits、TG kits、TC kits、HDL-C kits、LDL-C kits、GLU kits、FFA kits	Nanjing Jiancheng Bioengineering Institute
Insulin ELISA kits、TNF-α ELISA kits、IL-6 ELISA kits 、IL-10 ELISA kits、MDA ELISA kits, CAT ELISA kits , SOD ELISA kits、 GSH-Px ELISA kits、CCK8 kits	Dalian Meilun Biotechnology Co., Ltd
Hematoxylin–eosin	Beijing Leagene Biotechnology Co., Ltd
oil red O、Oleic acid (OA) 、sodium palmitate (PA) were purchased from Sigma-Aldrich	Sigma Aldrich (Shanghai) Trading Co., Ltd
Reverse transcription Kit、PCR kits	Takara Biomedical Technology (Dalian) Co., Ltd.
DMEM medium	Thermo Fisher Scientific
Primer sequence	Sangon Biotech (Shanghai) Co., Ltd.

Table S2 Primer information of the V3-V4 region of the bacterial 16S rRNA gene

Sequencing region	Primer name	Sequence
V3+V4	806R	5'-GGACTACHVGGGTWTCTA-3'
	515F	5'-GTGCCAGCMGCCGCGGTAA-3'

Table S3. Primer sequences of animal experiment.

Gene	Sequences
GADPH	F：CATGGCCTTCCGTGTTCCTA<br>R：GCGGCACGTCAGATCCA
ACC1	F：GAACGGCCACTACGACAAAT<br>R：CTGCAGGTTCTCAATGCAAA
FAS	F：GCTGTGCTTGCAGCTTACTG<br>R：CGGATCACCTTCTTGAGAGC
PPARα	F：GCAGCTCTGACAGGTCATCA<br>R：ACTGCCGTTGTCTGTCACTG
PPARγ1	F：TCTCCATGACAGACATGGACA<br>R：GTCAGGCTGTTGGTCTCACA
PPARγ2	F：GGGTGAAACTCTGGGAGATTCTC<br>R：TCAGCAACCATTGGGTCAG
SCD1	F：TTCTTACACGACCACCACCA<br>R：CAGCCGAGCCTTGTAAGTTC
SREBP-1c	F：ATCTGTTGTAAGGTGTATTTGCTGGCTTGG<br>R：CTTCGCCTATGCTGGTGCACAGAGATGACT
IL-1β	F：CAACCAACAAGTGATATTCTCCATG<br>R：GATCCACACTCTCCAGCTGCA
IL-6	F：TAGTCCTTCCTACCCCAATTTCC<br>R：TTGGTCCTTAGCCACTCCTTC
TNF-α	F：ACGTGGAACTGGCAGAAGAG<br>R：TCACCCCGAAGTTCAGTAGA

Table S4 Primer sequences of cell experiment

Gene	Sequences
GADPH	F：TGCACCACCAACTGCTTAGC<br>R：GGCATGGACTGTGGTCATGAG
ACC1	F：GCTGCTCGGATCACTAGTGAA<br>R：TTCTGCTATCAGTCTGTCCAG
FAS	F：GAAACTGCAGGAGCTGTC<br>R：CACGGAGTTGAGGCGGAT
PPARα	F：ATGGTGGACACGGAAAGC<br>R：TTAATCGTCCTCTACGAC
PPARγ1	F：GAAATGACCATGGTTGAC<br>R：GATGCAGGCTCCACTTTG
PPARγ2	F：ATGGGTGAAACTCTGGGAGAT<br>R：GATGCAGGCTCCACTTTG
SCD1	F：CCTCTACCTGGAAGACGACATTCGC<br>R：GCAGCCGAGCTTTGTAAGAGCGGT
SREBP-1c	F：ACGGCAGCCCCTGTAACGACCACTGTGA<br>R：TGCCAAGATGGTTCCGCCACTCACCAGG
IL-6	F：GGCACTGGCAGAAAACAACC<br>R：GCAAGTCTCCTCATTGAATCC
IL-1β	F：CACGATGCACCTGTACGATCA<br>R：GTTGCTCCATATCCTGTCCCT
TNF-α	F：CCTGCCCCAATCCCTTTATT<br>R：CCCTAAGCCCCCAATTCTCT

Table S5 Non-alcoholic fatty liver disease activity score (NAS) scoring criteria

Severity score	Steatosis	Inflammation	Ballooning
0	≤5%	None	None
1	5%-33%	< 2	Rarely
2	33%-66%	2-4	Common
3	> 66%	> 4	——

Table S6 Statistical table of the number of different metabolites in each group

Differential metabolite grouping information	Number of significantly different metabolites	Down-regulate the number of metabolites	Up-regulate the number of metabolites
Control VS Model	278	163	115
Control VS GOS	260	172	88
Control VS Metformin	145	60	85
GOS VS Model	103	21	82
GOS VS Metformin	193	1	192
Metformin VS Model	135	112	23

Table S7 OD values of each group stained with oil red O (*n* = 6, mean±SEM)

Groups	OD value
Control	0.29±0.007***
FFA	0.59±0.015
GOS-L	0.49±0.005*
GOS-M	0.42±0.024**
GOS-H	0.33±0.019***

Note：vs FFA group， *p<0.05,**p<0.01,***p<0.001.
